# A hybrid approach to the simultaneous eliminating of power-line interference and associated ringing artifacts in electrocardiograms

**DOI:** 10.1186/1475-925X-12-42

**Published:** 2013-05-14

**Authors:** Xiaolin Zhou, Yuanting Zhang

**Affiliations:** 1 Institute of Biomedical and Health Engineering, Shenzhen Institutes of Advanced Technology, Chinese Academy of Sciences, Xili Nanshan, Shenzhen 518055, China; 2 Department of Electronic Engineering, The Chinese University of Hong Kong, Shatin, NT, Hong Kong

## Abstract

**Background:**

The second-order, infinite impulse response notch filter is widely used to remove electrical power line noise in electrocardiograms (ECGs). However this filtering process often introduces spurious ringing artifacts in the vicinity of raw signal with sharp transitions. It is challenging to simultaneously remove these two types of noise without losing vital information about cardiac activities.

**Objective:**

Our objective is to devise a method to remove the power-line interference without introducing artifacts nor losing vital information. To this end we have developed the "hybrid approach" involving two-sided filtration and multi-iterative approximation techniques. The two-sided filtration technique can suppress the interference but some cardiac components are lost. The lost information can be restored using multi-iterative approximation technique.

**Results:**

For evaluation, four artificial data sets, each including 91 ECGs of different heart rates, were generated by a dynamical model. Four publicly-accessible sets of clinical data (MIT-BIH Arrhythmia, QT, PTB Diagnostic ECG, and T-Wave Alternans Challenge Databases) were also selected. Our new hybrid approach and the existing method were tested with these two types of signal under various pre-determined conditions. In contrast with the existing method, the hybrid approach can provide more than 27.40 dB and 37.77 dB reduction in signal distortion for 95% and 60% of artificial ECGs respectively; it can provide in excess of 11.78 dB and 17.48 dB reduction in distortion for 95% and 60% of these real records respectively.

**Conclusions:**

Overall, a significant reduction in signal distortion is demonstrated. These test results indicate that the newly proposed approach outperforms the traditional method assessed on both the artificial and clinical ECGs and suggest it could be of practical use for clinicians in the future.

## Background

Biopotential signals, such as electrocardiogram (ECG), often suffer from power-line interference (PLI, 50 or 60 Hz) since the recorded signal is an output of the electric fields of coupling states surrounding main power lines (PLs) and the power of the body. PLI is probably the most common problem encountered in the processing of biopotential signals. Essentially, a notch filter is adapted for minimizing PLI because of its ability to reject narrow band noise. Indeed, the second-order, infinite impulse response (IIR) notch filter is routinely applied for this purpose [1-3]. Because of the transient response effect of the notch filter, the impulse response of this type filter generally has an oscillatory behavior, which may cause microvolt-level ringing artifacts (RAs, typically ranging between 0 and 40 μV) in the immediate regions of input signal with sharp transitions. Besides, it will cause undesirable attenuation in signal components at frequencies close to the center frequency (50 or 60 Hz). Tolerable signal distortion needs a narrow stopband bandwidth (SBW); however, a narrower SBW results in a longer transient response time (TRT); whilst a longer TRT often incurs more serious RAs. It is an inherent contradiction. When an ECG signal is being processed, the RAs occur in the right side of QRS complexes, and consequently, this implies that many cardiac components are lost in ST-T regions. Serious distortion (signal distortion caused by the SBW itself and the appreciable RAs) may make the ECG signal more difficult to interpret, particularly for the ST-T segment analysis, QT interval estimation, the detection of Ventricular Late Potentials (VLPs) and so on [4-6]. Removal of PLI however should be done with utmost stringent efforts not to eliminate or distort the raw signals without introducing artifacts nor losing vital information [7-9]. Many sophisticated digital methods have been investigated to cope with either 50 or 60 Hz interference [10-12], and they satisfy the requirement for suppression and even elimination of PLI during ECG signals acquisition. However, it is impossible to design an IIR notch filter to remove PLI without causing distortion [13-15], and this problem is still unsolved in practice. In this paper we address the challenges to simultaneously remove the PLI and RAs without losing critical cardiac components by developing a new method which we call the "hybrid approach".

Being motivated by early pioneering work on investigating the RAs phenomena caused by the suppression of PLI, in this paper, the hybrid approach comprising two-sided filtration and multi-iterative approximation techniques, is proposed to simultaneously minimizing the PLI and associated RAs. In the first instance, the two-sided filtration technique is partitioned into four steps, which are applied to eliminate the PLI, localize the RAs and remove them afterward, whilst handling the boundary effects which are caused by the practical causal filter. To deal with the inherent contradictions, next the multi-iterative approximation technique is accomplished in three steps, which are adopted to sequentially reconstruct the lost cardiac information. A combination may thus prove to be more effective in eliminating these two types of noise. The elaborate scheme of the hybrid approach is stated in Sect. 3.

From the practical viewpoints of industrial and clinical applications, a bio-model-oriented diagnostic signal processing technique should be evaluated on the clinical data that are acquired from numerous subjects, as well as the artificial data that cover a variety of specific pre-determined conditions. Using artificial ECGs has a typical advantage, in that the signal distortion can be precisely calculated, since the ideal signal (i.e., "true" signal) can be reset to any desired case. In this study, the performances of the proposed and existing methods are evaluated in detail with artificial ECGs which are generated by an open-source program [16,17], as well as four well-used, clinical ECG databases. All of these real data are accessible to the public at Physionet [18]. We compared these two methods with respect to signal distortion in the absence and presence of artificial PLs, respectively. We also examined noise reduction in the presence of artificial PLs. Specifically, these data sets are classified into four groups: with and without the addition of artificial electrical PLs under the notch filter with the center frequency of 50 and 60 Hz, respectively. The first two groups that without PLs, are designed for quantitatively investigating the signal distortion. The other two groups, which mixed with the artificial PLs, are chosen for evaluating the distortion in an environment with interference, together with examining the capacities of the newly presented and old methods for removing PLI. The relationship of SBW and TRT of notch filters has not been well delineated, to provide more insight in the next section we also detail the key properties of finite impulse response (FIR) and IIR notch filters, so that their properties can be compared with each other. Additionally, related RAs are quantitatively examined, and the challenges are outlined.

### Problem statement

A digital notch filter is a band-stop filter that passes all frequency components except those lying within a narrow range centered on a center frequency *f*_0_. The magnitude response of an ideal notch filter may be given as below,

(1) $$\left|H_{d}\left(e^{\mathrm{j\omega}}\right)\right|=\left\{\begin{array}{c} 1,\omega \neq \omega_{0}\\ 0,\omega =\omega_{0} \end{array}\right)$$

where *ω* = 2*πf*/*f*_*s*_ is the normalized digital frequency, *ω*_0_ = 2*πf*_0_/*f*_*s*_ is the normalized center frequency at *f*_0_. *f*_*s*_ is the sampling rate, and *f* is the specified frequency. In practice, the notch filter has a SBW at *f*_0_, that is, Δ*ω* = 2*π*Δ*f*/*f*_*s*_. Δ*ω* and Δ*f* are normalized and digital SBWs, respectively.

#### FIR and IIR notch filters

Let *H*_*f*_(*z*) denote the transfer function of a second-order, FIR notch filter,

(2) $$H_{f}\left(z\right)=1-2\gamma z^{-1}+z^{-2}$$

where *γ* = cos *ω*_0_. *H*_*f*_(*z*) is simple and easy to implement. However, a disadvantage in using this kind filter is that the SBW of *H*_*f*_(*z*) is relatively large, which could not meet the specifications [19]. In order to be applicable at narrow SBW situations, a *H*_*f*_(*z*) based second-order, IIR notch filter is then commonly used [3,20],

(3.a) $$H_{i}\left(z\right)=\beta_{0}\cdot \frac{H_{f}\left(z\right)}{1-2\gamma \beta_{0}z^{-1}+\left(1-\lambda \right)\beta_{0}z^{-2}}$$

where *β*_0_ = 1/(1 + *λ*) and *λ* = tan(Δ*ω*/2). We regulate 0 < *λ* < 1  in this study. Eq. (3.a) can be rearranged as follows,

(3.b) $$H_{i}\left(z\right)=\frac{b_{0}+b_{1}z^{-1}+b_{2}z^{-2}}{a_{0}+a_{1}z^{-1}+a_{2}z^{-2}}$$

where *a*_0_ = 1, *b*_0_ = *b*_2_ = *β*_0_, *a*_1_ = *b*_1_ = − 2*γβ*_0_, and *a*_2_ = (1 − *λ*)*β*_0_. First provided *γ* ≠ − 1, *H*_*i*_(*z*) contains two poles (*α*_1_ and *α*_2_) inside the unit circle |*z*| = 1 at *z* complex plane,

(4) $$\alpha_{1,2}=\beta_{0}\cdot \left(\gamma \pm \sqrt{\gamma^{2}+\lambda^{2}-1}\right)$$

where *α*_1_ + *α*_2_ = − *a*_1_, *α*_1_ ⋅ *α*_2_ = *a*_2_. The stability and settling time of *H*_*i*_(*z*) are characterized by these two poles.

Let *x*[*n*] and *y*[*n*] be the input and output signals at discrete time *n*, respectively. This filter can be implemented by the following difference equation,

(5.a) $$y\left[n\right]=-\overset{2}{\underset{k=1}{\sum }}a_{k}y\left[n-k\right]+\overset{2}{\underset{k=0}{\sum }}b_{k}x\left[n-k\right]$$

where the subscript *k* refers to the *k*th-order index of *H*_*i*_(*z*). By deduction, in essence, Eq. (5.a) can be identified as follows,

(5.b) $$y\left[n\right]=\overset{\infty }{\underset{k=0}{\sum }}h\left[k\right]x\left[n-k\right]$$

where *h*[*k*] represents the impulse response of *H*_*i*_(*z*) (see Appendix A),

(6) $$h\left[k\right]=\;\left\{\begin{array}{r} b_{0},\;k=0\\ \;\left(-a_{1}\right)h\left[k-1\right]+b_{1},\;k=1\\ \left(-a_{1}\right)h\left[k-1\right]+\left(-a_{2}\right)h\left[k-2\right]+b_{2},\;k=2\\ \vdots \\ \;\left(-a_{1}\right)h\left[k-1\right]+\left(-a_{2}\right)h\left[k-2\right],\;k\geq 3 \end{array}\right)$$

*H*_*i*_(*z*) is a stable system, since |*a*_2_| < 1 and |*a*_1_| < 1 + *a*_2_. When *H*_*i*_(*z*) contains a pair of complex valued poles or a single negative pole, *h*[*k*] will cycle back and forth between negative and positive during the transient state [21], which indicates that *h*[*k*] is associated with an oscillatory behavior.

**Figure 1:**
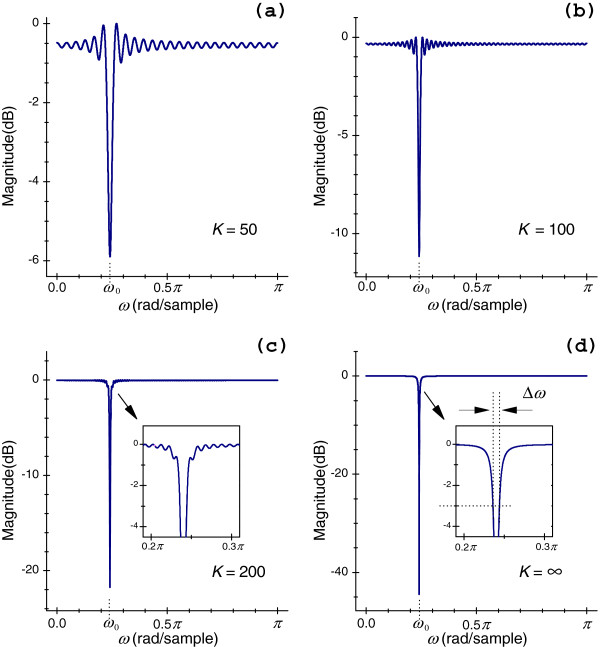
**Effects of different values of *K*.** (**a**) FIR filter with *K* = 50; (**b**) FIR filter with *K* = 100; (**c**) FIR filter with *K* = 200; (**d**) IIR filter with *K* = *∞*; (**a**)-(**c**) are calculated by Eq. (6), respectively. (**d**) is calculated by Eq. (3.b), which is equivalent to Eq. (5.b). These notch filters are designed at *f*_*s*_ = 500 Hz with *f*_0_ = 60 Hz, and Δ*f* = 2.0 Hz.

Recalling Eq. (5.b), let us consider a finite case, this can be written as,

(7) $$y\left[n\right]=\overset{K}{\underset{k=0}{\sum }}h\left[k\right]x\left[n-k\right]$$

where *K* is a positive integer. One interpretation of Eq. (7) is that it represents a FIR notch system. Figure 1(a)-(d) display FIR and IIR notch filters calculated by Eqs. (6) and (3.b), respectively. Notably, the higher order *K* of the filter, the weaker intensity the pass-band ripples and greater the selectivity. Consulting Figure 1(a)-(d), it is clear that this kind FIR filter has the following limitations: (A) The order *K* is considerably higher than that of an equivalent second-order IIR filter meeting the same requirements. It thus has far more computational complexity. (B) Because of many pass-band ripples, signals that include the information of interest inside the relevant frequency bands will be grossly distorted. This is an issue related to the pseudo-Gibbs phenomena.

#### Inherent contradictions

**Figure 2:**
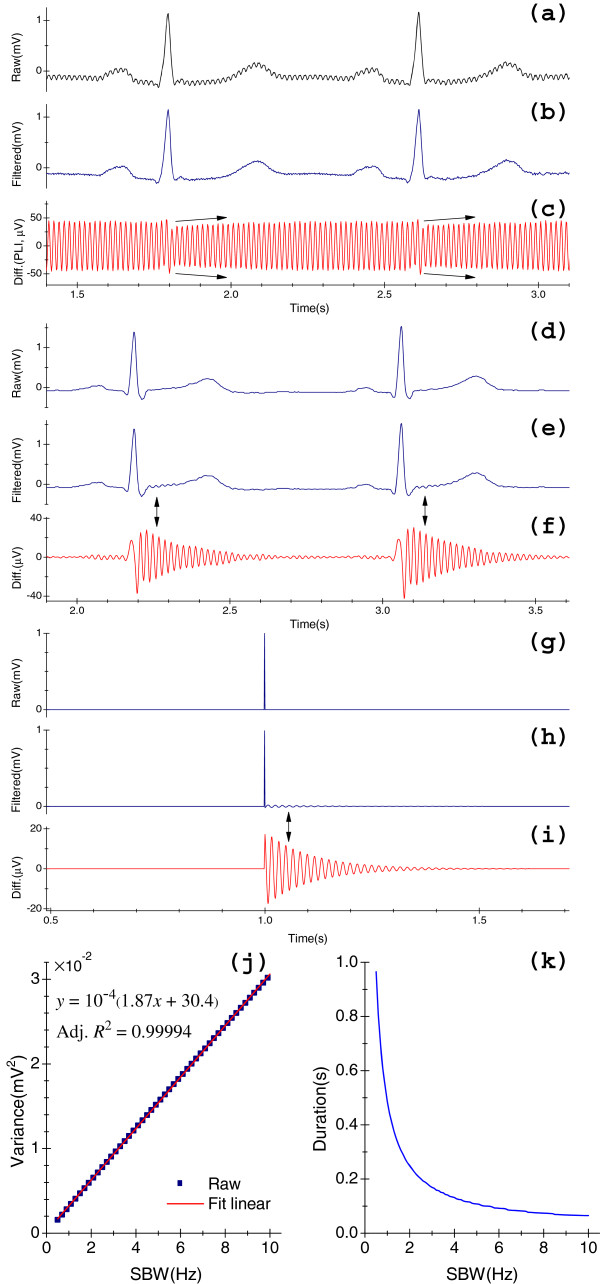
**Results of processing of notch filters *H***_***i ***_**(*z*).** (**a**) Clinical ECG with 50 Hz real PLI; (**b**) The output of this kind filter when applied to the signal in (**a**); (**c**) Differentiated components of (**a**) and (**b**); (**d**) Clinical ECG without PLI; (**e**) The output of the signal in (**d**) after passing it to this kind filter; (**f**) Differentiated components of (**d**) and (**e**); (**g**) A simulated unit impulse signal (1 mV); (**h**) The output of *H*_*i*_(*z*) when applied to the signal in (**g**); (**i**) Differentiated results of (**g**) and (**h**); (**j**) The distributions of $\sigma_{v}^{2}$ at various SBW; (**k**) The distributions of duration of RAs at various SBW.

The *H*_*i*_(*z*) is often used for removing PLI. Figure 2(a)-(c) display an input clinical ECG that is corrupted by real PLI, the output of the input signal after passing it to *H*_*i*_(*z*) and the relevant residue portion, respectively. In Figure 2(b) we see that PLI is well canceled. Ideally, PLI should be eliminated without any undesirable effects to distort the raw signals. Unfortunately, this cannot be achieved completely. RAs can sometimes interfere with the ST-T regions. The *H*_*i*_(*z*) operates with IIR of oscillation, which probably distorts the input as well as attenuates the amplitude by producing RAs. Consequently, important cardiac components will be lost. In contrast to the ECG signal, RAs are not remarkable. In other words, they may pollute the ST segments (1 to 20 μV) [8], but they are not distinguishable by the naked eye unless the intensity of which is greater than a threshold, such as 1 μV. Just as in Figure 2(b), it seems as if there were no RAs. However, if we carefully check Figure 2(c) (as indicated by arrows), we find that they still exist.

Sharp transitions of signal may generate noticeable and intolerable RAs in the immediate vicinities of these abrupt changes. Figure 2(d)-(f) is another example, in which we clearly see the spurious effect of RAs: RAs with the amplitude up to 30 μV, as shown in Figure 2(f). Therefore, the RAs should be carefully removed to prevent the distortion of input signal. The main cause of RAs is due to the abrupt bandstop of *H*_*i*_(*z*), spectral components that lie within the Δ*f*, as well as those close to *f*_0_ ± Δ*f*/2, will be attenuated; this is the frequency-domain description. In the time domain, the cause of RAs is *H*_*i*_(*z*) itself: infinite impulse and oscillatory responses.

In order to quantitatively investigate the relationship between abrupt discontinuities of input signal and the corresponded RAs, we begin with the unit impulse signal, which is,

(8) $$\delta \left[n\right]=\left\{\begin{array}{c} 1,n=n_{0}\\ 0,n\neq n_{0} \end{array}\right)$$

where *n*_0_ is a specific time. For simplifying the mathematics that follows the processing, by definition, *δ*[*n*] starts at 0 (*n*_0_ = 0), and goes to *∞*. By making *δ*[*n*] pass through the system *H*_*i*_(*z*), and letting *y*_*f*_[*n*] be the output, *ν*[*n*] = *δ*[*n*] − *y*_*f*_[*n*] the difference. The variance of *ν*[*n*] is then calculated by,

(9.a) $$\begin{array}{c} \sigma_{v}^{2}=\overset{\infty }{\underset{n=0}{\sum }}v{\left[n\right]}^{2}=\overset{\infty }{\underset{n=0}{\sum }}{\left(\delta \left[n\right]-\overset{\infty }{\underset{k=0}{\sum }}h\left[k\right]\delta \left[n-k\right]\right)}^{2}\\ \;={\left(1-h\left[0\right]\right)}^{2}+\overset{\infty }{\underset{n=1}{\sum }}h{\left[n\right]}^{2}\\ \;=1-2h\left[0\right]+\overset{\infty }{\underset{n=0}{\sum }}h{\left[n\right]}^{2} \end{array}$$

By deduction (see Appendix B), we relate the $\sigma_{v}^{2}$ and *λ*,

(9.b) $$\sigma_{v}^{2}=\frac{1}{1-\lambda^{2}}\cdot \lambda ,\left(\sigma_{v}^{2}\propto \Delta f\right)$$

In most practical applications, provided *γ*^2^ + *λ*^2^ − 1 ≤ 0 (i.e., tan(Δ*ω*/2) ≤ sin *ω*_0_, commonly, Δ*ω*/2 ≪ *ω*_0_), then the pole radius *ρ* for *H*_*i*_(*z*) is given by,

(10) ρ=α1=α2=a2=1−λ1+λ,Ifγ2+λ2−1<0,Imα1=−Imα2≠0Ifγ=−1−λ2,α1=α2<0,ρ∝1Δf

Referring to Eqs. (9.b) and (10), we come to the overall conclusions: (*i*) If *λ* ≪ 1, $\sigma_{v}^{2}\;$ can be loosely interpreted as a linear function of Δ*f*. With Δ*f* increasing, more and more signal components in the stop and pass bands will be modified in both the amplitude ("ripple") and the phase. (*ii*) Eq. (10) expresses that when Δ*f* → 0, then *ρ* → 1. In other words, the wider the Δ*f*, the closer the location of poles to the origin in the *z* plane, meaning the system *H*_*i*_(*z*) settles more rapidly (also meaning the system has a shorter duration of TRT) [22]. Therefore, it indicates that the duration of RAs tends to decrease as Δ*f* increases. It is an inherent contradiction of notch filters.

To visualize this problem, next we use Eq. (8) to generate a 10-second-length signal that is digitized at 1000 Hz, as shown in Figure 2(g). For convention, let the impulse occur at *n*_0_ = 1 s (amplitude of the impulse is 1 mV). By making Δ*f* with an increment of 0.1 Hz from 0.5 to 10.0 Hz, we calculated the outputs *y*_*f*_[*n*] by Eq. (5.a) at *f*_0_ = 50 Hz with each Δ*f*. We thus obtained 96 outputs. For each output, at a specified Δ*f*, the duration of RAs is defined as the time from impulse to the point *w*, at which,

(11) $$\overset{w}{\underset{n=0}{\sum }}{\left(\delta \left[n\right]-y_{f}\left[n\right]\right)}^{2}/\left(\frac{\lambda }{1-\lambda^{2}}\right)\geq 95\%$$

Figure 2(h) shows the output at Δ*f* = 3.0 Hz, and Figure 2(i) displays the corresponding residual components. Figures 2(j) and (k) plot all the calculated results. Observe that these results agree with the aforementioned conclusions.

### The principle of this algorithm

As we have seen, a cardinal implication is how the QRS complex affects the output of system *H*_*i*_(*z*); to the system it always poses a big impulse. Because *H*_*i*_(*z*) is a causal filter, noticeably time-decaying RAs can only occur on the right side of QRS complexes, see Figures 2(e), (f), (h) and (i). This implies that RAs only depend on the input waveforms regardless of whether the output waveforms which are of steep transitions or not. In addition, Eqs. (9.b) and (10) offer insights on the determinants of *H*_*i*_(*z*), which are uniquely controlled by its Δ*f*. These key intrinsic properties of *H*_*i*_(*z*) would be applied in this newly developed approach.

As before, *x*[*n*] denotes the input, the number of samples is *L* and *x*^**T**^[*n*] denotes the counterpart of the signal *x*[*n*], that is, *x*^**T**^[*n*] = *x*[*L* − 1 − *n*]. Then we construct a mirror extended signal,

(12) $$x_{\mathrm{me}}\left[n\right]=\left\{\begin{array}{l} x\left[n\right],\;0\leq n\leq L-1\\ x^{T}\left[n-L\right],\;L\leq n\leq 2L-1 \end{array}\right)$$

We explore two-sided filtration and multi-iterative approximation techniques to eliminate the probable PLI and RAs which are contained in *x*_*me*_[*n*]. For the various filter parmeters, let $y_{\mathrm{me}}^{r}\left[n\right]\;$ denote outputs of the signal *x*_*me*_[*n*] after passing it to the systems *H*_*i*_(*z*), $y_{\mathrm{me}}^{n}\left[n\right]\;$ denote outputs of this new method when applied to *x*_*me*_[*n*].

#### Two-sided filtration technique

The design procedure can be summarized as follows:

Step 1 - Initialization

Given *f*_*s*_ and *f*_0_, at a specified Δ*f*, we use Eq. (3.b) to calculate filter coefficients *a*_1_, *a*_2_, *b*_0_, *b*_1_ and *b*_2_. *bCons* = ⌊ *f*_*s*_/*f*_*b*_ ⌋, ⌊·⌋ represents a round operator, *f*_*b*_ = 125 Hz is a constant. *bCons* is an integer that is chosen to accommodate different *f*_*s*_. If *bCons* < 2, let*bCons* = 2.

Step 2 - Twice notch filtering

(*i*) First notch filter suppressing. We pass the *x*_*me*_[*n*] to the notch filter, that is the Eq. (5.a) with filter coefficients calculated in the previous step, and get the output $y_{\mathrm{me}}^{r}\left[n\right]$ as the system output. Then we can obtain the differential components *dy*_*me*_[*n*] using the derivative filter followed,

(13) $$dy_{\mathrm{me}}\left[n\right]=x_{\mathrm{me}}\left[n\right]-y_{\mathrm{me}}^{r}\left[n\right]$$

**Figure 3:**
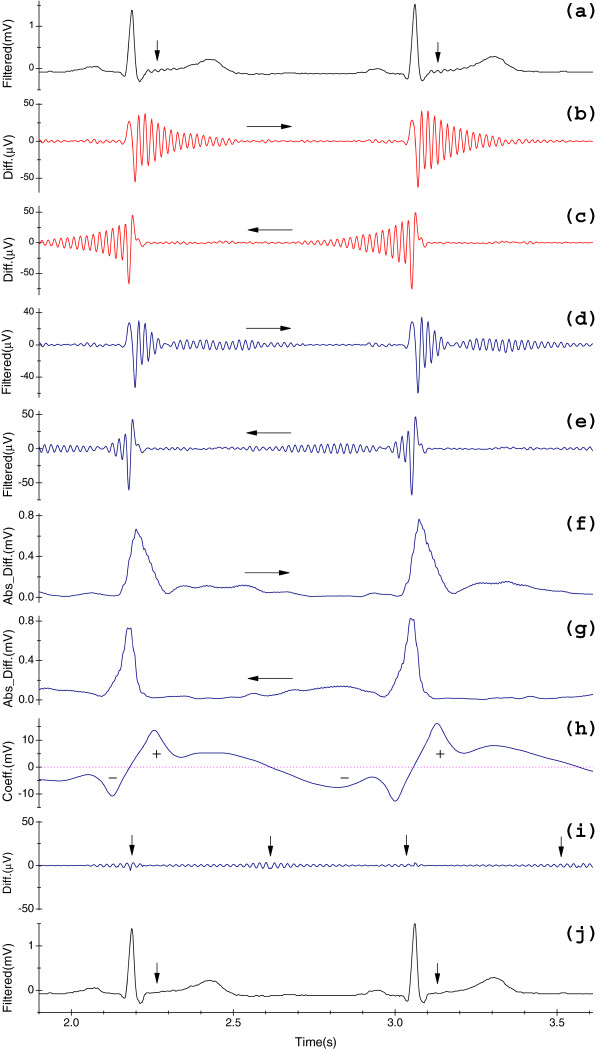
**Schematic diagram of the two-sided filtration technique.** (**a**) The output $y_{\mathrm{me}}^{r}\left[n\right]\;$ of the notch filter *H*_*i*_(*z*) when applied to the signal in Figure 2(d); (**b**) First half of *dy*_*me*_[*n*]; (**c**) Another half of *dy*_*me*_[*n*]; (**d**) First half of $dy_{\mathrm{me}}^{'}\left[n\right]$; (**e**) Second half of $dy_{\mathrm{me}}^{'}\left[n\right]$; (**f)** First half of *ls*_*me*_[*n*]; (**g**) Second half of *ls*_*me*_[*n*]; (**h**) Segment of *js*_*me*_[*n*]; (**i**) Residues $x_{\mathrm{me}}\left[n\right]-y_{\mathrm{me}}^{n}\left[n\right]$; (**j**) The ultimate output $y_{\mathrm{me}}^{n}\left[n\right]$ of the signal in Figure 2(d) processed by the present method. (**c**), (**e**) and (**g**) show results at relevant positions of the first halves. To this example, it is implemented at *f*_*s*_ = 500 Hz with *f*_0_ = 50 Hz, and Δ*f* = 3.0 Hz.

Figure 3(a) illustrates *y*_*me*_^*r*^[*n*] with the lost cardiac components, as indicated by the arrows. By means of signal-mirror extension, for a single filtering of *x*_*me*_[*n*], it includes two operations: one is conducted in *x*[*n*] in the forward direction, see Figure 3(b), which shows the first half of *dy*_*me*_[*n*] (0 *≤ n ≤ L *– 1 ); the other is conducted in the counterpart *x*^T^[*n*], which is equivalent to be operated in *x*[*n*] in the backward direction, as displayed in Figure 3(c), which shows the next half of *dy*_*me*_[*n*] (*L ≤ n ≤ *2*L* – 1) at relevant positions of the first half. The "relevant positions" means a sample at *n* is corresponded to the sample at n* (n* = 2*L* – 1 – *n*), hereinafter the same. Because *H*_*i*_ (*z*) is a causal system, detectable RAs in both halves of *dy*_*me*_[*n*] lie on right and left arms of the same QRS complexes, respectively.

(*ii*) Second notch filter suppressing. Sequentially, let *dy*_*me*_[*n*] be filtered by Eq. (5.a) with the same filter, we obtain the output $dy_{\mathrm{me}}^{'}\left[n\right]$. *dy*_*me*_[*n*] may involove the PLI, RAs and distorted components which are caused by the boundary effect [3]. However, $dy_{\mathrm{me}}^{'}\left[n\right]$ can only contains the major information of RAs and distorted portions. Figure 3(d) shows the first half of $dy_{\mathrm{me}}^{'}\left[n\right]$ and Figure 3(e) illuminates the second half of $dy_{\mathrm{me}}^{'}\left[n\right]$. In order to localize RAs in the next step, we need the second notch filtering operation. In fact, the possible PLI is directly suppressed in this step.

Step 3 - RAs localization

Let us first construct a sequence *cs*_*me*_[*n*] implemented by the derivative operation as below,

(14) $$cs_{\mathrm{me}}\left[n\right]=\left|dy_{\mathrm{me}}^{'}\left[n\right]-dy_{\mathrm{me}}^{'}\left[n-\mathrm{bCons}\right]\right|$$

the time delay of Eq. (14) is *bCons*/2 samples. Next we let *cs*_*me*_[*n*] pass through a low-pass filter shown as follows, and let *ls*_*me*_[*n*] denote as the relevant output,

(15) $$H_{l0}\left(z\right)=\frac{1-z^{-\mathrm{lOrder}}}{1-z^{-1}}$$

where *lOrder* = 4 · *bCons*, intrinsic delay of *H*_*l*0_(*z*) is (*lOrder* − 1)/2 samples and the gain is *lOrder*. For extracting the information of RAs, this low-pass filter is introduced to filter out fluctuations around the input sample and suppress undesirable outliers. Figure 3(f) displays the first half of *ls*_*me*_[*n*] and Figure 3(g) shows the second half. From Figures 3(f) and (g), it is clear that triangle-like spikes localize the RAs.

Step 4 - Determination of the output

Likewise, we construct another sequence *ds*_*me*_[*n*], which results from the following operation,

(16) $$ds_{\mathrm{me}}\left[n\right]=ls_{\mathrm{me}}\left[n\right]-ls_{\mathrm{me}}\left[n^{*}\right]$$

The use of Eq. (16) makes it possible to distinguish the RAs by means of positive and negative samples of *ds*_*me*_[*n*]. Due to the same consideration in *Step 3*, we let *ds*_*me*_[*n*] be processed by another low-pass filter defined as below,

(17) $$H_{l1}\left(z\right)=\frac{1-z^{-\mathrm{jOrder}}}{1-z^{-1}}$$

where *jOrder* = 16 · *bCons*, the phase delay of *H*_*l*1_(*z*) is (*jOrder* − 1)/2 samples and gain is *jOrder*, and let *js*_*me*_[*n*] denote the output. Figure 3(h) shows the first half of *js*_*me*_[*n*], and we see Figures 3(b) and (c), it is obvious that to each sample at position  *n*: (1) if *js*_*me*_[*n*] > 0, it represents that it gathers the information of RAs that lie within one arm of QRS complexes (left or right); (2) if *js*_*me*_[*n*] < 0, it means that it gather the information of RAs that lie within the other arm of QRS complexes (right or left), see Figures 3(b), (c) and (h). Therefore, to each sample, we eliminate RAs based upon the criteria as below,

(i) If *js*_*me*_[*n*] < 0, $y_{\mathrm{me}}^{n}\left[n\right]=y_{\mathrm{me}}^{r}\left[n\right]+dy_{\mathrm{me}}^{'}\left[n\right]$;

(ii)  If *js*_*me*_[*n*] > 0, $y_{\mathrm{me}}^{n}\left[n\right]=y_{\mathrm{me}}^{r}\left[n^{*}\right]+dy_{\mathrm{me}}^{'}\left[n^{*}\right]$;

(iii)  If *js*_*me*_[*n*] = 0 and *ls*_*me*_[*n*] < *ls*_*me*_[n*], *y*^*n*^_*me*_[*n*] = *y*^*r*^_*me*_ [*n*] + *dy*^*'*^_*me*_[*n*];

(iv)  Otherwise, $y_{\mathrm{me}}^{n}\left[n\right]=y_{\mathrm{me}}^{r}\left[n^{*}\right]+dy_{\mathrm{me}}^{'}\left[n^{*}\right].$

Furthermore, another benefit of criteria (*i*)-(*iv*) is avoiding the boundary effect. Figure 3(j) shows the output $y_{\mathrm{me}}^{n}\left[n\right]\;$ processed by this new method, and Figure 3(i) shows the residue portion $x_{\mathrm{me}}\left[n\right]-y_{\mathrm{me}}^{n}\left[n\right]$. Pay special attention to the details of Figures 3(a) and (j), and see the regions with arrows in Figure 3(j), then consult Figures 3(b), (c) and (i), we easily find that RAs are mostly eliminated. Note worthily, variables *bCons*, *lOrder* and *jOrder* are self-adaptive with respect to *f*_*s*_. Although the introduction of both-sided filtration increases the memory requirements and the proposed method also increases the complexity by introducing logical tests in *Step 4*, two-sided filtration restores the phase distortions caused by the one-sided operation.

#### Multi-iterative approximation technique

The single implementation of previous technique has two minor drawbacks, both of which are presented in Figure 3(i): (1) it is based upon the assumption that RAs consist of non-overlapping portions at positions of both directions of *x*[*n*]. In fact, it would not be able to distinguish overlapped RAs. However, *H*_*i*_(*z*) of a small Δ*f* may incur some appreciable parts (greater than 1 μV) overlapped in the middle of adjacent QRS complexes, as shown by the second and forth arrows in Figure 3(i). (2) Another major limitation encountered in using such a practical filter is that the system will attenuate waveforms constituted by high frequencies surrounding *f*_0_, as we see from positions with first and third arrows in Figure 3(i). As in the former disadvantage, intuitively, we might apply the *H*_*i*_(*z*) with a larger Δ*f* to overcome this. From Eq (9.b), however, such a filter may cause more attenuation in signal components at frequencies close to *f*_0_, since the duration of RAs and the amount of lost components are mutually dependent upon each other.

A novel method that may overcome the fundamental problems is to repeat the processing of two-sided filtration several times with various Δ*f*; we name it as multi-iterative approximation technique. Specifically, it can be partitioned into ternary steps as below,

Step I - Denoising with a large reference Δ*f*. In order to make distinctive RAs do not overlap each other, the *H*_*i*_(*z*) with a large enough Δ*f* should be adapted. Whereas, it does not denote that the larger the Δ*f*, the better the performance. Consulting Eqs. (9.b) and (10) and Figures 2(j), (k), it is clear that duration of RAs tends to decrease drastically as Δ*f* increases at small Δ*f* cases; but it decreases slightly at large Δ*f* cases. Furthermore, $\sigma_{v}^{2}$ is roughly linear with respect to the Δ*f*. Thus, an appropriate compromise should be made to fit the task: an empirical and experimental Δ*f* = 6.0 Hz is employed here.

**Figure 4:**
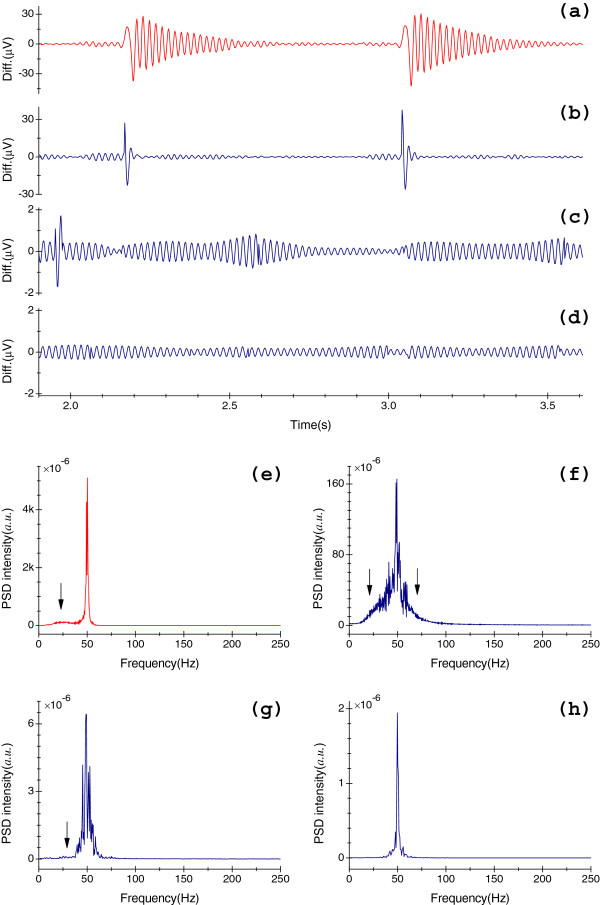
**Comparison of residual coefficients and relevant PSD spectra.** (**a**) The $x_{\mathrm{me}}\left[n\right]-y_{\mathrm{me}}^{r}\left[n\right]$ results from the old method (Δ*f* = 2.0 Hz); (**b**) Residual part $x_{\mathrm{me}}\left[n\right]-y_{\mathrm{me}}^{n}\left[n\right]$ in *Step I* (Δ*f* = 6.0 Hz); (**c**) Residual part $y_{\mathrm{me}}^{d}\left[n\right]$ in *Step II* (Δ*f* = 2.0 Hz); (**d**) Residual part $y_{\mathrm{me}}^{s}\left[n\right]$ in *Step III* (Δ*f* = 2.0 Hz); (**e**)-(**h**) show PSD spectra of sequences in (**a**)-(**d**), respectively. Spectra of (**e**)-(**h**) are calculated by Welch's method [26] at *f*_*s*_ = 500 Hz.

Input *x*_*me*_[*n*] is depicted in Figure 2(d). Figure 4(b) illustrates the residual part $x_{\mathrm{me}}\left[n\right]-y_{\mathrm{me}}^{n}\;\left[n\right]$, which is obtained at Δ*f* = 6.0 Hz, and Figure 4(f) shows the related PSD spectrum. In the time domain, certain amounts of components of QRS complexes have been obviously lost, as we can see from Figure 4(b). It can likewise be seen in Figure 4(f) that parts of the signal of interest, which are close to *f*_0_, have been suppressed because of this large Δ*f* in the frequency domain.

Step II - Reconstruction of details with a small target Δ*f*. In *Step I*, we achieve a short duration of RAs, and thus RAs can be eliminated. However, because of the large SBW Δ*f*, many cardiac components are lost. Thereby, in addition to possible PLs, residual part $x_{\mathrm{me}}\left[n\right]-y_{\mathrm{me}}^{n}\left[n\right]$ also contains lost components of original signal. In order to extract these useful details, $x_{\mathrm{me}}\left[n\right]-y_{\mathrm{me}}^{n}\left[n\right]$ is then to be processed by the two-sided filtration technique with a small Δ*f* (Δ*f* < 6.0 Hz). Let $x_{\mathrm{me}}^{d}\left[n\right]=x_{\mathrm{me}}\left[n\right]-y_{\mathrm{me}}^{n}\left[n\right]$ denote the input, $y_{\mathrm{me}}^{d}\left[n\right]$ the output.

*Step I* and *Step II* are complementary with respect to Eqs (9.b) and (10). To illustrate, Figure 4(c) shows the $y_{\mathrm{me}}^{d}\left[n\right]$ at Δ*f* = 2.0 Hz and Figure 4(g) displays the relevant PSD spectrum of $y_{\mathrm{me}}^{d}\left[n\right]$. It can be observed from Figures 4(b), (c), (f) and (g) that most details have been reconstructed.

Step III - Reconstruction of slight details with the same target Δ*f*. We can see the vicinities indicated by arrows in Figure 4(g), $y_{\mathrm{me}}^{d}\left[n\right]$ may still contain a little valuable information, just as the results shown in Figure 4(c): the amplitude of which is greater than 1 μV. To further reduce the distortion effects, similarly, let $x_{\mathrm{me}}^{s}\left[n\right]=x_{\mathrm{me}}^{d}\left[n\right]-y_{\mathrm{me}}^{d}\left[n\right]$, $x_{\mathrm{me}}^{s}\left[n\right]$ is then be processed by the two-sided filtration technique with the same Δ*f* in *Step II*. Let $y_{\mathrm{me}}^{s}\left[n\right]$ as the output. Figures 4(d) and (h) show $y_{\mathrm{me}}^{s}\left[n\right]$ and the relevant spectrum, respectively. We see that spectrum content in pass bands of Figure 4(h) is flatter than that of Figure 4(g), this represents that most lost details have been reconstructed, since the amplitude of $y_{\mathrm{me}}^{s}\left[n\right]$ is substantially less than 1 μV as shown in Figure 4(d).

The ultimate output signal is derived from,

(18) $$y_{\mathrm{me}}^{n}\left[n\right]=x_{\mathrm{me}}\left[n\right]-\left(x_{\mathrm{me}}^{s}\left[n\right]-y_{\mathrm{me}}^{s}\left[n\right]\right),\left(n\in \left[0,L-1\right]\right)$$

Figure 4(a) displays the residual part $x_{\mathrm{me}}\left[n\right]-y_{\mathrm{me}}^{r}\left[n\right]$ obtained by the old method, and the relevant PSD spectrum is illustrated in Figure 4(e). Observe that many cardiac components, which lie in the pass bands, have been removed, as illustrated at positions with the arrow in Figure 4(e). By this new method, lost components have been minimized substantially in both time and frequency domains, as shown in Figure 4(a)-(d) and Figure 4(e)-(h), respectively.

**Figure 5:**
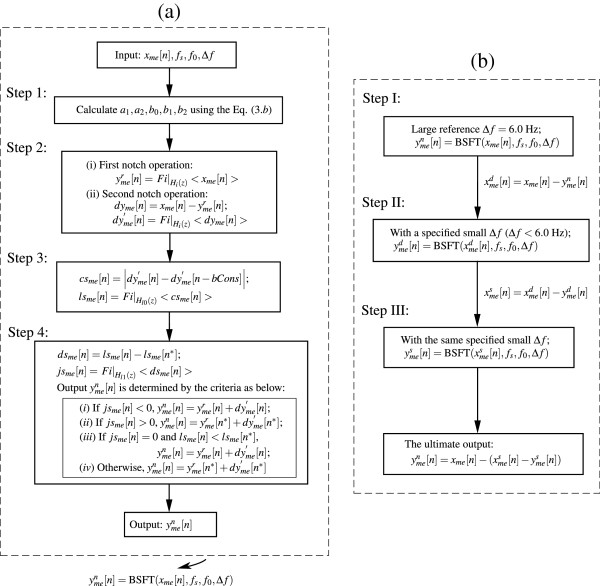
**Flowcharts of the newly proposed algorithm.** (**a**) Flowchart of the two-sided filtration technique; (**b**) Flowchart of the multi-iterative approximation technique. *Y*_*out*_[*n*] = *Fi|*_H(z)_ <X_in_[n]>indicates that the input X_in_[n] is filtered by the system H(z), and the relevant output is *Y*_*out*_[*n*]. y^n^_me_[n] = **BSFT** (x_*me*_[n], f_s_, f_0_, Δf) indicates that the input x_*me*_[n] is processed by the Two-sided filtration technique with the specified parameters f_s_, f_0_ and Δf, and the relevant output is y^n^_me_[n].

Although one should aim to fully restore the lost components, it should be noted at this point that the residual coefficients $y_{\mathrm{me}}^{s}\left[n\right]$ may still contain a certain amount of cardiac components which fall within this SBW Δ*f* (i.e., [*f*_0_ − Δ*f*/2, *f*_0_ + Δ*f*/2]); consequentially, these components can never be reconstructed due to the so-called "frequency overlap", as we can see from Figure 4(d). It is an inherent weakness of *H*_*i*_(*z*). Figure 5 shows the flowcharts of the newly proposed algorithm; Figure 5(a) depicts the flowchart of two-sided filtration technique; Figure 5(b) represents the flowchart of multi-iterative approximation technique. Notably, Δ*f* is the only parameter that needs to be specified in this method.

### Materials and evaluation

#### Artificial and clinical ECG data sets

We simulated four artificial ECG data sets with *f*_*s*_ of 250, 360, 500 and 1000 Hz, respectively. The generator generates realistic ECGs with user-settable parameters, such as "sampling frequency", "internal sampling frequency" and so on. This generator can be accessed from [16,17]. For this study, we used the generator to simulate 10-second-duration data at "internal sampling frequency" of 2000 Hz (but 720 Hz for ECGs sampled at 360 Hz) with the specified "mean heart rates", and regulated other parameters with default values [17]. To assess the performance of these two methods in an environment with various sharp transitions, to each data set, we simulated ECGs with the "mean heart rates" ranging from 50 to 140 beats per minute (BPM), in increments of 1 BPM. We thus obtained 91 data for each data set.

Four widely used sets of real ECGs (MIT-BIH Arrhythmia Database [MITDB], QT Database [QTDB], PTB Diagnostic ECG Database [PTBDB], and the T-Wave Alternans Challenge Database [TWADB]) were also selected for evaluation [18], see Table 1 for details. All of these data were tested in this study.

**Table 1:** Four publicly-accessible sets of clinical data are selected for evaluation

**Databases**	***f***_***s***_ **(Hz)**	**Data numbers/Channels (Durations)**	**Brief description**
QTDB	250	105/2-lead	It was chosen to represent a wide variety of QRS and ST-T morphologies.
		(15 min.)	
MITDB	360	48/2-lead	It was obtained from 47 subjects and contains affluent arrhythmia information.
		(30 min.)	
TWADB	500	100/multi-lead	Including subjects with risk factors, such as myocardial infarctins, transient ischemia, ventricular and so on, as well as healthy subjects.
		(2 min.(appro.))	
PTBDB	1000	549/15-lead	It was collected from 290 healthy volunteers and patients.
		(1.92 min.(appro.))	

#### Performance metrics

The main power supply is not perfectly stable. In some countries, tolerance of the frequency variation of PLs is 1% [23]. In poor electrical environments, however, the variation may up to 3% [24]. Because the bandwidth of real PLs is varying, in order to assess the performance of this method under different situations, to both clinical and artificial data, we conducted these tests by changing Δ*f* of the *H*_*i*_(*z*) from 1.0 to 4.0 Hz with an increment of 0.1 Hz, respectively. In addition, to determine the capacity of eliminating PLI, artificial PLs that were simulated by sinusoidal functions (with the amplitude of 0.1 mV) at industrial frequencies (50 and 60 Hz, respectively), were added to the raw data. To each lead, at a specified Δ*f*, different situations can be categorized into four groups: ❶ At *f*_0_ = 50 Hz, implement *H*_*i*_(*z*) without PLI; ❷ At *f*_0_ = 60 Hz, implement *H*_*i*_(*z*) without PLI; ❸ At *f*_0_ = 50 Hz, implement *H*_*i*_(*z*) with the addition of 50 Hz PLI; ➍ At *f*_0_ = 60 Hz, implement *H*_*i*_(*z*) with the addition of 60 Hz PLI.

We evaluated the relative distortion produced by two methods. The old method means the signals were only filtered by the *H*_*i*_(*z*). To each input signal *x*[*n*], we obtained the outputs *y*^*r*^[*n*] and *y*^*n*^[*n*] by processing input signal *x*[*n*] with the IIR filter, that is Eq. (5.a) and this new method, respectively. We calculated the relevant ratio of percentage root-mean-square difference (*rPRD*, in units of decibels [*dB*]), and it is given by,

(19) $$\mathrm{rPRD}=10\log{\mathrm{PRD}}_{r}^{2}-10\log{\mathrm{PRD}}_{n}^{2}=10\log\frac{\sigma_{r}^{2}}{\sigma_{n}^{2}}$$

where $\mathrm{PR}D_{r}^{2}=\frac{\sigma_{r}^{2}}{Am^{2}}$, $\mathrm{PR}D_{n}^{2}=\frac{\sigma_{n}^{2}}{Am^{2}}$, $Am^{2}=\overset{L-1}{\underset{n=0}{\sum }}x{\left[n\right]}^{2}$, $\sigma_{r}^{2}=\overset{L-1}{\underset{n=0}{\sum }}{\left(x\left[n\right]-y^{r}\left[n\right]\right)}^{2}$ and $\sigma_{n}^{2}=\overset{L-1}{\underset{n=0}{\sum }}{\left(x\left[n\right]-y^{n}\left[n\right]\right)}^{2}$. *rPRD* illustrates, for the new method, how distortion of the input *x*[*n*] is quantitatively lessened in comparison with the old method. It is not feasible to access the purely clinical signals, since these real signals might already contain PLs and other broadband noises. We hence let these signals be processed via the new approach first, and let the resulting signals be the inputs *x*[*n*] here.

To each data set with a specified group, wherein first, for the results *rPRD*s calculated from all of the various SBWs, we can figure out a threshold, at which, those results that, in excess of a certain percentage of total *rPRD*s, are greater than this threshold. Let *rPRD*|_95*%*_ and *rPRD*|_60*%*_ denote the thresholds with percentages of 95% and 60%, respectively. To facilitate comparisons with the old method in examining the capability of minimization of distortion at a specific SBW, then, for those results *rPRD*s outputted by the same SBW Δ*f*, at which (i.e., this single Δ*f*), we can define $\mathrm{rPRD}{|}_{50\%}^{\mathrm{\Delta f}}$ denote the threshold with a percentage of 50%. Therefore, *rPRD*|_95*%*_, *rPRD*|_60*%*_ and $\mathrm{rPRD}{|}_{50\%}^{\mathrm{\Delta f}}$ represent the overall performances of this newly presented method.

## Results and discussion

**Figure 6:**
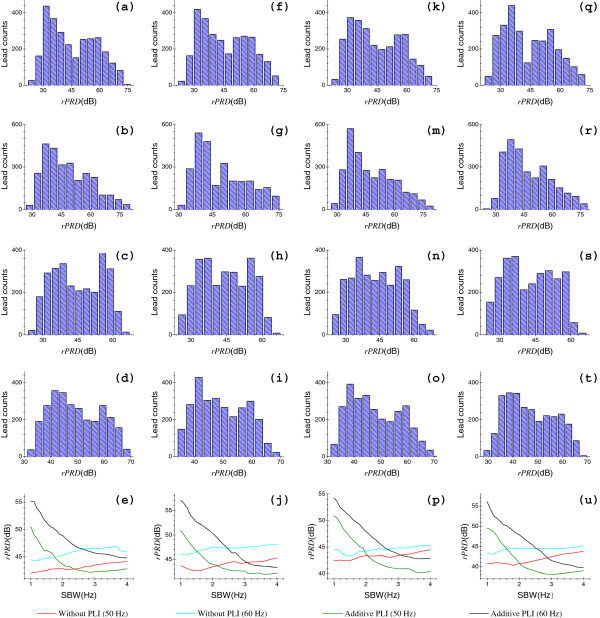
**Histograms and relevant statistical results of artificial data sets.** From left to right: the four columns, (**a**)-(**e**), (**f**)-(**j**), (**k**)-(**p**) and (**q**)-(**u**), show results of data sets with *f*_*s*_ of 250, 360, 500 and 1000 Hz, respectively. For each data set, from top to bottom: the first four rows show results of four groups, including groups ❶, ❷, ❸ and ➍, respectively. Four curves in each inset in the last row, which contains (**e**), (**j)**, (**p**) and (**u**), correspond to four histograms in the same column and ordered by the legends, respectively.

**Figure 7:**
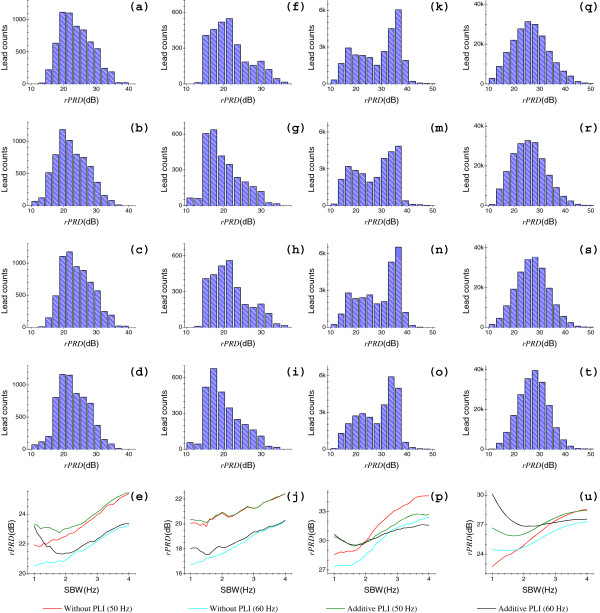
**Histograms and relevant statistical results of real data sets.** From left to right: the four columns, (**a**)-(**e**), (**f**)-(**j**), (**k**)-(**p**) and (**q**)-(**u**), show results of data sets with *f*_*s*_ of 250, 360, 500 and 1000 Hz, respectively. For each data set, from top to bottom: the first four rows show results of four groups, including groups ❶, ❷, ❸ and ➍, respectively. Four curves in each inset in the last row, which contains (**e**), (**j**), (**p**) and (**u**), correspond to four histograms in the same column and ordered by the legends, respectively.

Test results of the artificial and clinical data sets are shown in Figures 6 and 7, respectively; where each histogram illustrates the results of a data set for a specific group. For each artificial data set of a specific group, we have 2821 results calculated by Eq. (19). For a specific group, we have 6510, 2976, 28892 and 204228 results for QTDB, MITDB, TWADB and PTBDB, respectively. Insets, in the last rows of Figures 6 and 7, display the statistical results ($\mathrm{rPRD}{|}_{50\%}^{\mathrm{\Delta f}}$) corresponding to the histograms in the first four rows, respectively; for a specific group with varying Δ*f*, each curve is associated to a histogram in the same column. Tables 2 and 3 give the statistical results (*rPRD*|_95*%*_ and *rPRD*|_60*%*_) corresponding to the histograms in Figures 6 and 7, respectively.

**Table 2:** Statistical results of all of the four artificial ECG data sets

***f***_***s***_ **(Hz)**	**Without PLI**				**Additive PLI (0.1 mV)**			
	***f***_**0**_** = 50 Hz(−❶-)**		***f***_**0**_** = 60 Hz(−❷-)**		***f***_**0**_** = 50 Hz(−❸-)**		***f***_**0**_** = 60 Hz(−➍-)**	
	***rPRD*|**_**95%**_**(dB)**	***rPRD*|**_**60%**_**(dB)**	***rPRD*|**_**95%**_**(dB)**	***rPRD*|**_**60%**_**(dB)**	***rPRD*|**_**95%**_**(dB)**	***rPRD*|**_**60%**_**(dB)**	***rPRD*|**_**95%**_**(dB)**	***rPRD*|**_**60%**_**(dB)**
250	28.82	38.82	33.20	42.53	29.49	40.25	35.93	45.29
360	28.91	38.75	34.76	42.60	29.67	40.48	36.86	45.60
500	28.09	38.60	33.01	41.05	27.88	39.20	34.66	43.68
1000	27.40	37.77	32.70	41.19	27.62	38.12	33.78	42.69

**Table 3:** Statistical results of all of the four clinical ECG data sets

***f***_***s***_**(Hz)**	**Without PLI**				**Additive PLI (0.1 mV)**			
	***f***_**0**_** = 50 Hz(−❶-)**		***f***_**0**_** = 60 Hz(−❷-)**		***f***_**0**_** = 50 Hz(−❸-)**		***f***_**0**_** = 60 Hz(−➍-)**	
	***rPRD*|**_**95%**_**(dB)**	***rPRD*|**_**60%**_**(dB)**	***rPRD*|**_**95%**_**(dB)**	***rPRD*|**_**60%**_**(dB)**	***rPRD*|**_**95%**_**(dB)**	***rPRD*|**_**60%**_**(dB)**	***rPRD*|**_**95%**_**(dB)**	***rPRD*|**_**60%**_**(dB)**
250^a^	17.05	21.86	15.07	20.58	17.46	22.14	16.02	21.10
360^b^	15.25	19.78	11.78	17.48	15.29	19.90	12.24	17.71
500^c^	14.66	26.16	14.71	24.86	15.53	26.66	15.76	26.71
1000^d^	14.67	24.20	15.88	23.85	16.58	25.38	18.07	26.07

^a^QTDB. ^b^MITDB. ^c^TWADB. ^d^PTBDB.

Figure 6(a)-(e) and Figure 7(a)-(e) plot the results using the artificial data set and QTDB (*f*_*s*_ = 250 Hz), respectively. To groups ❶ and ❷, as Δ*f* increases, it tends to produce larger $\mathrm{rPRD}{|}_{50\%}^{\mathrm{\Delta f}}$ for both artificial and clinical data sets as a whole. By contrast, to both groups ❸ and ➍, as Δ*f* increases, it tends to produce $\mathrm{rPRD}{|}_{50\%}^{\mathrm{\Delta f}}$ with relationships that look like the exponential decay for artificial data sets, while relationships with $\mathrm{rPRD}{|}_{50\%}^{\mathrm{\Delta f}}$ decreasing first and then increasing for QTDB data as a whole. With regard to different notch frequencies, the performances are significantly different for both artificial and clinical ECG data sets, as we see from Figures 6(e) and 7(e). From Table 2, to four groups, the minimum *rPRD*|_95*%*_ and *rPRD*|_60*%*_ were 28.82 dB and 38.82 dB for artificial ECGs, respectively. Similarly, in all of four groups, the minimum *rPRD*|_95*%*_ and *rPRD*|_60*%*_ were 15.07 dB and 20.58 dB for QTDB ECGs as shown in Table 3, respectively. It is worth noting that QTDB data was chosen specifically to contain a broad variety of QRS morphologies (i.e., various kinds of abrupt discontinuities) [25]. At this point, the results of QTDB ECGs are relatively more objective in the present study.

Figures 6(f)-(i) and 7(f)-(i) display the results using the artificial data set and MITDB (*f*_*s*_ = 360 Hz), respectively. Figures 6(j) and 7(j) are the relevant $\mathrm{rPRD}{|}_{50\%}^{\mathrm{\Delta f}}$ relations of Figures 6(f)-(i) and 7(f)-(i), respectively. MITDB includes in excess of 53 leads with abnormal, wide size or low intensity QRS waveforms, which are of low frequency components (i.e., slow transitions or smooth variations). Therefore, this turns out that less distortion results from the old method, since RAs always occur surrounding the notch frequency *f*_0_, that is the high-frequency end in most ECG spectra. Hence, the performance of the new method is not very good for MITDB data, as illustrated in Figure 7(j). Likewise, with respect to the different notch frequencies, the performances differ markedly for both artificial and clinical ECG data sets, as we can see from Figures 6(j) and 7(j). However, for the same notch frequency (50 or 60 Hz, respectively), the performances are not significantly different for MITDB data without or with the addition of artificial PLs. Of these statistical results, as displayed in Tables 2 and 3, for four groups, the minimum *rPRD*|_95*%*_ and *rPRD*|_60*%*_ were 28.91 dB and 38.75 dB for artificial ECGs, respectively; for the QTDB recordings with four groups, the minimum *rPRD*|_95*%*_ and *rPRD*|_60*%*_ were 11.78 dB and 17.48 dB, respectively.

The statistical results of artificial data set and TWADB (*f*_*s*_ = 500 Hz) are shown in Figures 6(k)-(p) and 7(k)-(p), respectively. In terms of artificial ECG data with four groups, as Δ*f* increases, they exhibit consistent tendencies with these of artificial ECGs sampled at 250 and 360 Hz. However, for the TWADB data with four groups, as Δ*f* increases, the performances depict no significant consistency as those of QTDB and MITDB, since the $\mathrm{rPRD}{|}_{50\%}^{\mathrm{\Delta f}}$ are divergent at sides of low and large SBWs, while relatively convergent in the middle of SBWs. In addition, for the QTDB and MITDB data sets, results with high probabilities lie in low sides of SBWs (i.e., less than 22 dB), but for the TWADB data the results of high probabilities lie within high sides (i.e., greater than 30 dB). In Tables 2 and 3, for the four groups, we see that the minimum *rPRD*|_95*%*_ and *rPRD*|_60*%*_ were 27.88 dB and 38.60 dB, 14.66 dB and 24.86 dB, for artificial and TWADB data sets, respectively. Furthermore, from Figures 6(e), (j), (p) and Table 3, for this clinical data set, the performance of the new method is better than that of the QTDB and MITDB data sets as a whole.

It can be clearly seen from Figures 6(q)-(u) and 7(q)-(u) that, for the artificial data set and PTBDB (*f*_*s*_ = 1000 Hz), respectively, the results of the artificial ECGs reveal no significant difference compared with three earlier used artificial data sets. However, the clinical ECGs have significant differences in the histograms as well as the $\mathrm{rPRD}{|}_{50\%}^{\mathrm{\Delta f}}$ relations to these of three previous clinical ECGs. As far as the histograms of the four groups are concerned, they exhibit Gaussian probability distributions, since a huge number of results was obtained (a total of 204228 results) for each group, as shown in Figure 7(q)-(t). In terms of $\mathrm{rPRD}{|}_{50\%}^{\mathrm{\Delta f}}$ relations, as shown in Figure 7(u), four curves show tendencies toward convergence as Δ*f* increases. Again from Tables 2 and 3, the minimum *rPRD*|_95*%*_ and *rPRD*|_60*%*_ were 27.40 dB and 37.77 dB for artificial ECGs of four groups, respectively; and for the PTBDB recordings, the minimum *rPRD*|_95*%*_ and *rPRD*|_60*%*_ were 14.67 dB and 23.85 dB, respectively.

Generally, for the artificial ECGs of a specific group, differences of results which were obtained from different data sets, show no special significance, since the only differences of each data set are the sampling rates *f*_*s*_ and the duration *L*. Regarding the four clinical ECG data sets (each including four groups), however, they showed obviously different performances, since these data sets were obtained from various subjects and with different emphases. In particular, the MITDB recordings, comprising many and a broad variety of wide size QRS complexes, show relatively poor performance. From all the results of artificial and clinical ECGs sampled at each *f*_*s*_, we can find that the performance exhibited by the artificial data set is significantly better than that of the relevant clinical data set. The reason is that the clinical ECGs may contain many low frequency leads. In summary, all of these test results indicate that the proposed method has the capacity to well reduce the PLI, and simultaneously, greatly minimize the RAs for both artificial and clinical ECGs.

### Benefits and limitations

As previously mentioned, we always want the SBW of a notch filter to be very narrow for suppressing PLI. The FIR notch filter, which is defined by Eq. (7), does not encounter the "infinite impulse" problem, but it requires a large degree *K* to meet the specification, and this often comes at the cost of high computational complexity. Additionally, another problem with the FIR notch filter is that it may still produce RAs since it is oscillatory in the range of the "finite response", because *K* is a large number. In contrast, our hybrid approach provides primary benefits in eliminating specific interferences. It is not limited to reducing fixed fundamental PLI (50 or 60 Hz) but also applicable to removing the high-frequency harmonics for some worse cases, since the *f*_0_ can be tailored to any desired frequency in this approach.

Thus far, all of our discussions are based upon the tan(Δ*ω*/2) ≤ sin *ω*_0_. In certain applications, however, some rare cases may still exist where tan(Δ*ω*/2) > sin *ω*_0_, especially those ECG monitor systems with low sampling rates *f*_*s*_. In general, such situations occur at *f*_*s*_ = 2*f*_0_ + *δ*_*f*_ and 0 ≤ *δ*_*f*_ ≪ *f*_*s*_. By Eq. (4), consider the following two possible cases,

(i) *γ* = − 1 That is, *f*_0_ = *f*_*s*_/2. According to Eq. (3.a), *H*_*i*_(*z*) represents a first-order, IIR notch filter that has only one pole *α* inside the unit circle, for this case,

(20) $$\rho^{'}=\left|\alpha \right|=-\alpha =a_{2}=\frac{1-\lambda }{1+\lambda },\left(\alpha <0,\rho^{'}\propto 1/\Delta f\right)$$

Likewise, for the input *δ*[*n*], we can obtain the output variance,

(21) $$\sigma_{v}^{2^{'}}=\frac{1}{1-\lambda^{2}}\cdot \lambda ,\left(\sigma_{v}^{2^{'}}\propto \Delta f\right)$$

Eqs. (20) and (21) demonstrate similar forms with Eqs. (10) and (9.b), respectively; it implies that the hybrid approach is also applicable. However, real PLs own a frequency bandwidth, the counterparts of PLs that lie within the right side of *f*_0_ will pollute valuable information of low frequencies. It is a limitation not caused by the method but *f*_*s*_ itself. Therefore, we recommend *δ*_*f*_ ≥ 4.0 Hz for practical application based upon the current study and literature [23,24].

(ii) − 1 < *γ* < 0 and *γ*^2^ + *λ*^2^ − 1 > 0 That is, |*α*_1_| ≠ |*α*_2_|. This yields (see Appendix C),

(22) $$\begin{array}{c} \mathrm{Max}\left(\left|\alpha_{1}\right|,\left|\alpha_{2}\right|\right)=\left|\gamma \right|\cdot \frac{1+\sqrt{1-\gamma^{-2}+\gamma^{-2}\lambda^{2}}}{1+\lambda },\\ \left(\alpha_{1}<0,\alpha_{2}<0,\mathrm{Max}\left(\left|\alpha_{1}\right|,\left|\alpha_{2}\right|\right)\propto \Delta f\right) \end{array}$$

Max(|*α*_1_|, |*α*_2_|) denotes the larger one of |*α*_1_| and |*α*_2_|. For |*α*_1_| ≠ |*α*_2_|, the settling time of *H*_*i*_(*z*) is mainly determined by Max(|*α*_1_|, |*α*_2_|) [22]. Within this situation, using the *H*_*i*_(*z*) of a larger Δ*f* would not be able to achieve shorter durations of RAs but with more cardiac components lost. The "larger" reference Δ*f* is then set to the specified target Δ*f* in *Step I* to meet the uniformity within the approach. Thus, it is worth emphasizing that more precautions should be taken when Δ*f* is adjusted to avoiding overlapping RAs in the middle of adjacent QRS complexes. Indeed, it is essential to remember this limitation as well, since Eq. (10) is not universal but with conditions.

## Conclusions

Instead of frequency response, specifications of second-order, IIR notch filter may be given in terms of the impulse response. In this study, we started with the impulse response of *H*_*i*_(*z*), the output $\sigma_{v}^{2}$ and the settling time that, concerning its behaviour in the time domain, have been quantitatively investigated. Minimizing the RAs involved in the signal of sharp transitions by removing PLI is a great concern in the processing of biopotential signal. The detection and analysis of the VLPs in the ECG signal is highly sensitive to the residual PLI and the RAs after the QRS complexes as a result of the filtering technique applied. The artifacts may become considerable in cases of high and steep complexes. Owing to the intrinsic properties of *H*_*i*_(*z*), we proposed a hybrid approach, which utilizes *H*_*i*_(*z*), to reliably suppress PLI as well as to aviod the generation of RAs. It is applicable to different *f*_*s*_ and easy to implement. In fact, Δ*f* is the only parameter that needs to be specified for this approach. Problems are greatly mitigated via these techniques. Sufficient results and performance statistics are provided to validate the reliability of this method in the test environment with a variety of conditions (e.g., artificial and clinical ECGs, of various *f*_*s*_, with altering Δ*f*, etc.). An eventual consideration related to practice is *f*_0_ of PLs. To the artificial PLs used in this study, *f*_0_ was set to 50 or 60 Hz without varying. However, *f*_0_ of real PLs, similar to its bandwidth, may fluctuate over a small range. Even so, we can refer to many previous studies for how to adaptive tracking of the *f*_0_ with serious drift.

## Appendix A: Proof of the Equation (6)

A real signal *x*[*n*] can be expressed as the sum of linear superposition of unit impulse functions *δ*[*n* − *k*],

(a.1) $$x\left[n\right]=\overset{\infty }{\underset{k=0}{\sum }}x\left[k\right]\delta \left[n-k\right]$$

where, if *k* = *n*, *δ*[*n* − *k*] = 1; otherwise, if *k* ≠ *n*, *δ*[*n* − *k*] = 0. By definition, to each impulse function *δ*[*n* − *k*], the output is denoted as the impulse response *h*[*n* − *k*]. Consulting Eq. (5.a), we obtain,

(a.2) $$\begin{array}{c} \;h\left[n\right]=-a_{1}h\left[n-1\right]-a_{2}h[n-2]\\ \;+b_{0}\delta \left[n\right]+b_{1}\delta \left[n-1\right]+b_{2}\delta [n-2] \end{array}$$

Provide *H*_*i*_(*z*) is a causal system, then we conclude that,

(i) If *n* = 0, *h*[*n* − 1] = *h*[*n* − 2] = 0. Ignore *δ*[*n* − 1] and *δ*[*n* − 2], since *δ*[*n* − 1] = *δ*[*n* − 2] = 0, then,

(a.3) $$\;h\left[n\right]=b_{0}$$

(ii)  If *n* = 1, because *h*[*n* − 2] = 0, and *δ*[*n*] = *δ*[*n* − 2] = 0, which results in,

(a.4) $$\;h\left[n\right]=\left(-a_{1}\right)h\left[n-1\right]+b_{1}$$

(iii)   If *n* = 2, *δ*[*n*] = *δ*[*n* − 1] = 0, we have,

(a.5) $$\;h\left[n\right]=\left(-a_{1}\right)h\left[n-1\right]+\left(-a_{2}\right)h\left[n-2\right]+b_{2}$$

(iv)   If *n* ≥ 3,  *δ*[*n*] = *δ*[*n* − 1] = *δ*[*n* − 2] = 0, this yields,

(a.6) $$\;h\left[n\right]=\left(-a_{1}\right)h\left[n-1\right]+\left(-a_{2}\right)h\left[n-2\right]$$

We thus obtain Eqs (5.b) and (6) in terms of the filter coefficients *a*_0_, *a*_1_, *a*_2_, *b*_0_, *b*_1_   and *b*_2_.

## Appendix B: Proof of the Equation (9.b)

The *v*[*n*] = *δ*[*n*] − *y*_*f*_[*n*]  is equivalent to the input *δ*[*n*] to be processed by the following comb filter,

(b.1) $$\;H_{c}\left(z\right)=1-H_{i}\left(z\right)=c_{0}\cdot \frac{1-z^{-2}}{a_{0}+a_{1}z^{-1}+a_{2}z^{-2}}$$

where *c*_0_ = *λβ*_0_. If *γ* ≠ − 1, *H*_*c*_(*z*) also contains two poles at *α*_1_ and *α*_2_ inside the unit circle |*z*| at *z* plane.

According to Parseval's theorem, $\sigma_{v}^{2}$ can be calculated by,

(b.2) $$\begin{array}{c} \sigma_{v}^{2}=\overset{\infty }{\underset{n=0}{\sum }}v{\left[n\right]}^{2}=\frac{1}{2\pi }\overset{\pi }{\underset{-\pi }{\int }}{\left|H_{c}\left(e^{\mathrm{j\omega}}\right)\right|}^{2}d\omega \\ \;=\frac{1}{2\mathrm{\pi j}}\oint_{c}\frac{H_{c}\left(z\right)H_{c}\left(z^{-1}\right)}{z}dz \end{array}$$

where ∮_c_ represents the integral taken around the unit circle in counter-clockwise direction, and let

(b.3) $$F\left(z\right)=\frac{H_{c}\left(z\right)H_{c}\left(z^{-1}\right)}{z}=-\frac{c_{0}^{2}}{z}\cdot \overset{2}{\underset{i=1}{\prod }}\frac{1-z^{2}}{\left(z-\alpha_{i}\right)\left(1-\alpha_{i}z\right)}$$

By the Residue theorem, we can obtain Eq. (b.4),

(b.4) $$\sigma_{v}^{2}=\overset{2}{\underset{k=1}{\sum }}\mathrm{Res}\left[F\left(z\right),\alpha_{k}\right]$$

By Eq. (b.3), we calculate residue values ξ and ζ at each pole,

(b.5) $$\left\{\begin{array}{c} \xi =F\left(z\right){|}_{z=\alpha_{1}}=-\frac{c_{0}^{2}}{\alpha_{1}}\cdot \frac{1-\alpha_{1}^{2}}{\left(\alpha_{1}-\alpha_{2}\right)\left(1-\alpha_{1}\alpha_{2}\right)}\;\\ \zeta =F\left(z\right){|}_{z=\alpha_{2}}=-\frac{c_{0}^{2}}{\alpha_{2}}\cdot \frac{1-\alpha_{2}^{2}}{\left(\alpha_{2}-\alpha_{1}\right)\left(1-\alpha_{1}\alpha_{2}\right)}\; \end{array}\right)$$

And so,

(b.6) $$\sigma_{v}^{2}=\xi +\zeta =c_{0}^{2}\cdot \frac{1+a_{2}}{a_{2}\left(1-a_{2}\right)}=\frac{1}{1-\lambda^{2}}\cdot \lambda$$

## Appendix C: Proof related to the Equation (22)

For convention, we define,

(c.1) $$\phi =\frac{1+\varphi }{1+\lambda }$$

where,

(c.2) $$\varphi =\sqrt{1-\gamma^{-2}+\gamma^{-2}\lambda^{2}}$$

Take the derivative of both sides of Eq. (c.2) with respect to *λ*, which results in,

(c.3) $$\Delta \varphi =\gamma^{-2}\varphi^{-1}\lambda \Delta \lambda$$

Then,

(c.4) $$\begin{array}{c} \;\Delta \phi =\frac{\left(1+\lambda \right)\Delta \varphi -\left(1+\varphi \right)\Delta \lambda }{\left(1+\lambda \right)\left(1+\lambda +\Delta \lambda \right)}\\ \;=\frac{\gamma^{-2}\varphi^{-1}\lambda \left(1+\lambda \right)-\left(1+\varphi \right)}{\left(1+\lambda \right)\left(1+\lambda +\Delta \lambda \right)}\Delta \lambda \\ \;=\frac{ℱ\left(\lambda \right)}{G\left(\lambda \right)}\Delta \lambda \end{array}$$

where,

(c.5) $$\left\{\begin{array}{c} \;ℱ\left(\lambda \right)=\gamma^{-2}\lambda \left(1+\lambda \right)-\varphi \left(1+\varphi \right)\\ \;G\left(\lambda \right)=\varphi \left(1+\lambda \right)\left(1+\lambda +\Delta \lambda \right)\; \end{array}\right)$$

First assume the ℱ (*λ*) ≤ 0 is true, from Eq. (c.5), this yields,

(c.6) $$\gamma^{-2}\lambda \left(1+\lambda \right)\leq \varphi \left(1+\varphi \right)$$

Solve Ineq. (c.6), we obtain (1 + *λ*)^2^ ≤ 0. Due to *λ* > 0, thus the assumption ℱ (*λ*) ≤ 0 is false, then ℱ (*λ*) > 0. Because *G*(*λ*) > 0, we therefore have,

(c.7) $$\frac{ℱ\left(\lambda \right)}{G\left(\lambda \right)}>0\Rightarrow \Delta \phi >0\Rightarrow \frac{1+\sqrt{1-\gamma^{-2}+\gamma^{-2}\lambda^{2}}}{1+\lambda }\propto \Delta f$$

## Competing interests

The authors declare that they have no competing interests.

## Authors’ contributions

XZ developed the algorithm and drafted the manuscript. YZ provided suggestions and comments. All authors read and approved the final manuscript.
